# enviPath – The environmental contaminant biotransformation pathway resource

**DOI:** 10.1093/nar/gkv1229

**Published:** 2015-11-17

**Authors:** Jörg Wicker, Tim Lorsbach, Martin Gütlein, Emanuel Schmid, Diogo Latino, Stefan Kramer, Kathrin Fenner

**Affiliations:** 1 Institute of Computer Science, Johannes Gutenberg University Mainz, Staudingerweg 9, 55128 Mainz, Germany; 2 Scientific IT Services, ETH Zürich, Weinbergstrasse 11, 8092 Zürich, Switzerland; 3 Department of Environmental Chemistry, Eawag, Überlandstrasse 133, 8600 Dübendorf, Switzerland; 4 Department of Environmental Systems Science, ETH Zürich, 8092 Zürich, Switzerland

## Abstract

The University of Minnesota Biocatalysis/Biodegradation Database and Pathway Prediction System (UM-BBD/PPS) has been a unique resource covering microbial biotransformation pathways of primarily xenobiotic chemicals for over 15 years. This paper introduces the successor system, enviPath (The Environmental Contaminant Biotransformation Pathway Resource), which is a complete redesign and reimplementation of UM-BBD/PPS. enviPath uses the database from the UM-BBD/PPS as a basis, extends the use of this database, and allows users to include their own data to support multiple use cases. Relative reasoning is supported for the refinement of predictions and to allow its extensions in terms of previously published, but not implemented machine learning models. User access is simplified by providing a REST API that simplifies the inclusion of enviPath into existing workflows. An RDF database is used to enable simple integration with other databases. enviPath is publicly available at https://envipath.org with free and open access to its core data.

## INTRODUCTION

enviPath is the successor of the University of Minnesota Biocatalysis/Biodegradation Database and Pathway Prediction System (UM-BBD/PPS). The UM-BBD/PPS was recently transferred to Eawag and renamed to EAWAG-BBD/PPS (available at http://eawag-bbd.ethz.ch/index.html). The system has been completely redesigned, reimplemented and therefore renamed enviPath (https://envipath.org). The design of the implementation is explained in Section 1 and Section 2 of the Supplementary Material. Currently, both versions are available; however EAWAG-BBD/PPS will be discontinued in the next year.

enviPath is a unique resource for microbial biotransformation pathways of primarily xenobiotic chemical compounds. Compared to existing databases for central metabolism pathways such as KEGG (1,2), enviPath focuses on chemicals that are structurally more complex and that are known or suspected environmental contaminants. The goal of enviPath is to provide information on enzyme-catalyzed reactions of these environmental contaminants, which is of value for chemical risk assessment, bioremediation applications, and analysis of contaminants and their transformation products (TPs) in the environment.

enviPath predicts microbial biotransformation reactions using a substructure search, a rule base and atom-to-atom mapping. The system is able to recognize organic functional groups in a compound and predict transformations through generalized biotransformation rules. The biotransformation rules are based on reactions in the enviPath database and/or in the scientific literature.

One of the main goals of enviPath is to improve the notoriously low selectivity of current pathway prediction systems. Rule-based systems, including EAWAG-PPS, have been shown to predict TPs observed in the environment fairly comprehensively (i.e. to display high sensitivity). However, those systems predict many irrelevant products that are not likely to occur under specific environmental conditions (i.e. to display low selectivity) (3). This low selectivity leads to combinatorial explosion (i.e. the prediction of many irrelevant products) when rules are iteratively applied to predict consecutive biotransformation reactions. We hypothesize that at least three major reasons explain the low selectivity of EAWAG-PPS:

- Given the structural diversity of xenobiotic chemicals, the available data remain still limited.
- Most biotransformation data in EAWAG-BBD stem mainly from pure culture or enrichment studies, which are of limited relevance for actual environmental situations. Moreover, study conditions are generally not annotated.
- EAWAG-BBD lacks half-life information, although this information could significantly improve prediction efforts.

enviPath has been designed as a more modern and flexible successor system of EAWAG-BBD/PPS, to extend the functionality of the system and to address the above-mentioned shortcomings.

Currently, enviPath contains all data in terms of reactions, compounds, pathways and transformation rules from the EAWAG-BBD. The core data are and will always be available without registration and released under a creative commons license. By core data, we currently refer to the EAWAG-BBD data. We intend to also provide free access to future data sets published or reviewed by us and to updates to the EAWAG-BBD data set. The access to data by other users of the system can be restricted, depending on the configuration set by the user. To address (i) above, the new system has been designed to support users to add their own data efficiently. Users can also register and limit the access to their data. Data can be submitted to be reviewed and then marked accordingly to distinguish between data submitted by untrusted sources and data reviewed by a group of experts. By adding the functionality for users to add their own data to the system, we intend to make it easier to extend the database by including the community in the process. Additionally, as a result, the data are organized in packages; hence packages can be created that reflect certain environmental or experimental conditions.

Beyond the data available in the previous system, we extend the database to store additional meta-data on the reactions and rules ((ii) and (iii)), e.g. environmental conditions or the source of the data (provenance). Furthermore, half-life information can now be stored for a specific set of conditions for each compound. By making the prediction specific to certain environmental and/or experimental conditions, the quality of the biotransformation pathway prediction can be potentially improved. Moreover, we implemented machine learning-based techniques to limit the combinatorial explosion of biotransformation predictions, which were previously published (4,5), but not yet included in the system.

The remainder of the paper is organized as follows. We first explain the data stored in the database, which has been imported from EAWAG-BBD. We then list the main features of the prediction engine, which predicts pathways from compounds based on transformation rules. Next, we explain the reviewing system that ensures the quality of the database, followed by the functionality of the user and group system implemented in enviPath. Finally, we present use cases that illustrate the functionality of the system and close with an overview of related work and conclusions.

## DATA

**Figure 1.**
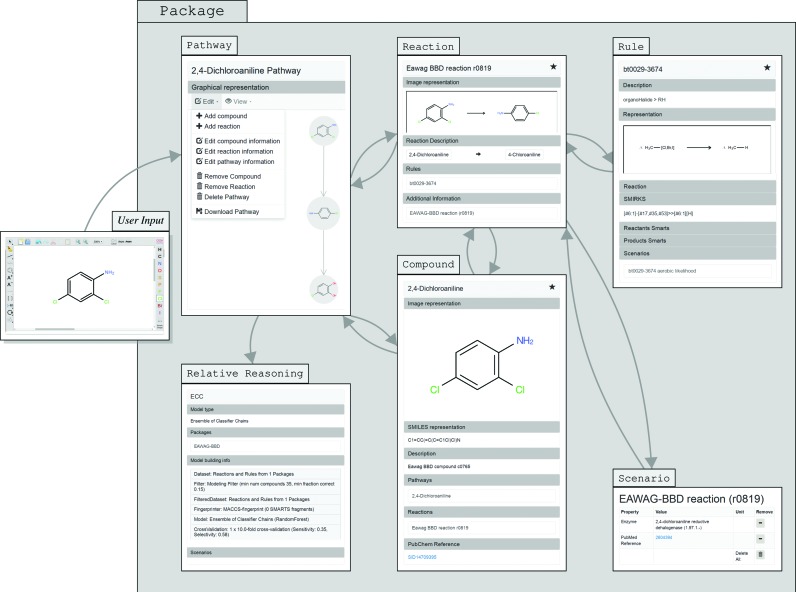
Diagram of the enviPath entities, the navigation possibilities between them and their display on screen (note that the figure does not display all logical relationships between the entities from the data model or a flow chart of the actual pathway prediction with inputs and outputs of computations). On the most left, user input is given via the web form in a visual editor or via SMILES input. From this input, a pathway is predicted using the Prediction Engine and truncation strategy. Alternatively, if a pathway for that compound is already stored, the stored pathway from the database is shown. Note that here we show an example from the database, not an actual predicted pathway. The pathway is linked to Reactions (on the edges) and Compounds (on the nodes). A Reaction is further linked to the Rule by which it is predicted. In the case of manually inserted Reactions, links to Rules can be present and set; in this case, the links explain from which Reaction the Rule is generalized. Furthermore, additional information is stored with all entities, named Scenario. An example is shown in the figure, in which the additional information of the Reaction, the link to the corresponding Pubchem entry and the enzyme involved in the reaction is displayed. Furthermore, every entity is organized in a Package.

enviPath contains all of the data from the former University of Minnesota Biocatalysis/Biodegradation Database UM-BBD, now EAWAG-BBD (http://eawag-bbd.ethz.ch), in a public package named ‘EAWAG-BBD’. EAWAG-BBD is a database that has been manually curated, primarily by the team of original developers of the UM-BBD (erem. Prof. L. B. M. Ellis and Prof. L. P. Wackett, both University of Minnesota, MN, USA), and contains information on over 1500 microbial catabolic reactions and approximately 220 biotransformation pathways. Additionally, reactions are associated with over 900 enzymes and more than 500 microorganisms. Currently, EAWAG-BBD has approximately 45 000 unique users per year. The majority of the data in EAWAG-BBD stems from pure culture or enrichment studies. EAWAG-BBD will be maintained in its current status and research teams are encouraged to submit pathways from pure culture or enrichment studies for review and inclusion into the package ‘EAWAG-BBD’. An overview of the data entities in the database and how they are connected is given in Figure 1.

A novel focus of our developer team will be on curating biotransformation pathway information that has been generated under more environmentally relevant conditions, i.e. in activated sludge, water-sediment systems and soil. This will include annotation of the experimental conditions under which the experiments were performed. As a first example, the package ‘DAR soil’ containing four exemplary pathways is presented in the current version of enviPath. ‘DAR soil’ contains pathway information from soil degradation studies extracted from pesticide registration dossiers (draft assessment reports, DAR) made publically available through the European Food Safety Authority (EFSA) (http://dar.efsa.europa.eu/dar-web/provision). In addition to the general biotransformation reaction scheme, these pathways contain information on different experimental conditions termed ‘scenarios’. Compounds in the pathway are associated with a given scenario when they have been observed under the specific experimental conditions. When available, a biotransformation half-life is additionally associated with a given compound in the pathway and a specific scenario. The package ‘DAR soil’ is planned to contain approximately 200 pesticide degradation pathways and more than 1000 biotransformation half-life values, with corresponding scenarios, by the end of 2016.

## PREDICTION ENGINE

The prediction engine of enviPath is based on EAWAG-PPS, and hence on its biotransformation rules (btrules), which have been imported into enviPath. Biotransformation rules are generalizations of reactions and used for the prediction of pathways. The left-hand side of a rule represents a pattern that matches functional groups in compounds. If the functional group is matched, then a transformation described via a mapping to the right-hand side of the rule is performed. An example of a transformation rule is given in Figure 4 in the Supplementary Material.

To represent the biotransformation rules, we use SMIRKS (see http://daylight.com/dayhtml_tutorials/languages/smirks/). enviPath can import or export transformation rules using this format. Additionally, the web interface offers a graphical molecule editor, Ketcher (6), that can be used to create transformation rules, which can be stored directly from the editor. enviPath uses AMBIT-SMARTS (7), which implements SMARTS and SMIRKS for CDK.

To predict a biotransformation pathway, the user submits a compound to the system. All transformation rules from the selected package(s) are applied to the compound. When a given rule is applicable, it is used to predict one or several products. Hence, a compound can have multiple products from this first step. The biotransformation rules are again applied to each product, and the process is repeated as long as products are predicted and terminated when a given stopping criterion is reached, e.g. a predefined number of prediction levels.

Notably, biotransformation rules must remain fairly general. These rules therefore get triggered quite easily; consequently, many transformation products are predicted that might not be relevant for a specific environmental condition. This process leads to large number of transformation products in each prediction cycle, and consequently, potentially to a combinatorial explosion. Thus, different methods were sought to limit the combinatorial explosion in pathway prediction.

In EAWAG-PPS, rule likelihoods were introduced to assess the aerobic likelihood of a transformation (8). Rules were categorized according to their aerobic likelihood into five categories: Very Likely, Likely, Neutral, Unlikely and Very Unlikely. The ranking into the likelihoods is an expert-based ranking, which was performed by participants of a series of UM-BBD workshops. Later, rule-based relative reasoning (9) was implemented to provide a possibility to prefer selected rules in the prediction (3). In this truncation strategy, the possibility to apply a rule depends on the presence of other applicable rules. Practically, this restriction requires additional (meta-)rules for the prioritization of rules and the resolution of conflicts. Recently, we developed a machine learning-based relative reasoning approach to estimate probabilities for each transformation (4,5). The aim of this method is to improve upon the low selectivity of rule-based relative reasoning. A visualization of the method is given in Figure 5 in the Supplementary Material. As the learning problem clearly is a multi-label classification problem (10), we extended the machine learning approach using multi-label classifiers to further improve the prediction (5).

The training of relative reasoning models on selected packages with new data can be used to reflect environmental conditions. The user simply assembles a package, either by entering new rules, pathways and reactions, or by combining entries from other packages. Then, a model can be trained on this package. The data in the package will be considered as ground truth, i.e. reactions predicted by the transformation rules that are not in this package will be considered as false.

All of these approaches, relative reasoning rules, single-label machine learning and multi-label machine learning, are included in enviPath, and we provide pre-trained relative reasoning models for the packages provided by us. The user can also train their own models on any collection of packages. Hence, new models can reflect exactly the conditions of the selected packages, and predicted probabilities are automatically adapted to the type of data used as input in the training process.

### Default settings

enviPath can be easily adapted to personal use cases. Users can change the packages used, and hence, the used transformation rules and models. Nevertheless, we included recommended settings, which are the default settings for users not registered at enviPath. The default settings are currently the data imported from EAWAG-BBD including all transformation rules and trained relative reasoning models on this data set. Because the machine learning-based models showed a better performance in our experiments, by default they are activated for the biotransformation pathway prediction.

## USER INTERACTION AND REVIEWING

One of the major differences of enviPath compared to its previous implementation, EAWAG-BBD/PPS, is its openness and flexibility. This system can be easily filled with data by every user or research group. Hence, enviPath can be considered as a wiki-like system because data can be modified given the right permissions. This flexibility provides the possibility for easier data collection because all users can easily submit data into the system and make the data publicly available.

Nevertheless, this creates a risk for the quality of the data because all users can submit random or not thoroughly curated data sets. Hence, we implemented a reviewing system that distinguishes curated data sets from not curated data. Whereas every user can submit data, all data are first marked as unreviewed. These data are hidden from the default users and the user must actively click on a link to view these data. When an owner of a package wants the data in that package to become reviewed and visible to the users, the owner can submit the package for review. After a review by a member of the group of reviewers, the package will be marked as reviewed. To ensure the quality of the reviewed data, the package becomes protected and can only be modified by the group of reviewers. Reviewers are a group of volunteers. At the moment, the authors are the only members of the reviewer group. Nevertheless, we plan to extend the group of reviewers to other trusted researchers. Through this extension, we plan to include the community in the process of collecting data and thereby broaden the basis of the system. A detailed workflow of the reviewing process is given in Figure 6 in the Supplementary Material.

## GROUPS AND USERS

enviPath introduces a sophisticated user and group management system. Whereas anonymous access remains possible and all basic functions are available without creating a user account, registered users have some advantages. Registered users can use the permission system of enviPath. Anonymous users can set permissions for packages as well, yet only registered users can set packages completely private, restricting access and modification privileges. Additionally, the user can grant reading or writing permissions to other users to collaboratively work on data in a package, e.g. the release of data related to a publication. Users can create packages with their researched pathways and later publish the data set on enviPath openly.

Collaboration with other users can be improved by establishing groups. Groups are collections of users and can be freely created. Permissions can be granted to groups in the identical way as they can be granted to users. In this way, research groups can be established as groups and collaborate on their data.

Another advantage of a registered user is the possibility to establish default settings for most operations, e.g. the list of packages to use for pathway prediction or browsing the database. By default, certain packages are selected for each user, but registered users can change this list and use different transformation rules for pathway predictions by default. Pathway prediction offers a wide variety of behavior, which are assigned a default setting for unregistered users and can be modified in each prediction. Registered users can change the default settings and do not have to modify them for each prediction.

### Anonymous access

Whereas the user registration at enviPath provides benefits with the usage, anonymous usage of the system is possible with restrictions. The most substantial restriction might be the limitation encountered when predicting pathways. The size of the predicted pathway is restricted because the prediction costs certain computing power. Currently, we restrict the number of predicted products to 50, which is a reasonably high number for this task. Nevertheless, depending on the computing resources available and the usage of the system, we might have to adapt this restriction. Similarly, whereas the registered user can at the moment use unlimited resources (limited only by the computing power of the server), we might have to add some restrictions in the future.

Another restriction for the anonymous user is the persistence of the data. All resources created by the anonymous user will be automatically deleted after 30 days. Data of registered users will not be deleted automatically.

## USE CASES

In this section, we describe some typical use cases of enviPath to motivate the functionality of the new features.

### Pathway prediction

The first use case is the standard use of the prediction engine, i.e. the prediction of a pathway by a user. To perform a prediction, the user has several options: First, the user can enter a SMILES or draw a structure using the molecule editor on the Home page and click the Go! button. This will start a prediction using all transformation rules that are set in the default settings with the standard settings for the prediction. The second option for the prediction of the pathway is to navigate to the Pathway section of the page and start the prediction using the entry in the menu on this page. In both cases, the resulting pathway will be stored in the default package of the user. The last option is to navigate to the Package section of the page, select a package and choose the Pathway section in the package. In this case, the pathway is created in the selected package.

In all three cases, the user is redirected to the page of the new pathway. Note, however, that depending on the complexity of the prediction, the prediction process might not be finished, i.e. levels of the pathway are not predicted yet. Nevertheless, a pathway will be shown to the user. This status is indicated by an animated loading symbol in the pathway view. If the prediction process is completed, then a ✓symbol is shown.

The moment the pathway prediction is started, the pathway is stored in the database. The system does not distinguish between predicted and manually created pathways, only between reviewed and unreviewed pathways. Each pathway is stored and can be accessed using a unique URI. The user can now send this URI to others to access the predicted pathway simply by sending the browser URL.

The pathway can later be modified and adapted using the editing symbols in the header line. Compounds and reactions can be added edited or deleted. Note that a delete will not delete the compound or reaction itself but only its presence in the pathway. The visualization of the pathway is automatically generated; hence any changes will not be stored in the database. However, the user can zoom in or out or move compounds that are currently not important for the user away by a simple drag and drop.

This use case can be extended when the user is registered. The user can give access to the pathway by adapting the permissions of the package and share all pathways in the package with a certain group of collaborators. In this case, the pathway can be set to be inaccessible by an anonymous user.

### Sharing of data

The second use case addresses the possibility of using enviPath as a database for environmental pathways. A research group can share data via enviPath simply by registering every member as a user. A group can be created in the Group interface. When the group is created, users can be given read permissions, indicating that they are simply members of the group, or write permissions, indicating that they themselves can add or remove users from the group and change the description of the group. Next, a package must be created in the Package section. In the package overview, permissions can be granted, and a write permission must be set to the previously created group.

Using the package, every member of the group can create data in the package using the corresponding links directly in the package. These data are immediately visible and writable for all members of the group.

Finally, when the group has filled the package with the data, the package can be made readable to the user anonymous, and hence to everyone. The package is then publicly accessible and can be referred to via the URL and easily downloaded.

## RELATED WORK

Other rule-based pathway prediction systems for microbial metabolism besides EAWAG-PPS include PathPred (11), Catalogic (12) and BNICE (13). Of those, PathPred and EAWAG-PPS are fully freely available, whereas Catalogic and BNICE are partially or fully commercial systems. The biotransformation rules in those systems, at least for Phase I metabolism, are to a large extent based on the respective data collected in the EAWAG-BBD (14). The existing systems rely on different approaches to limit combinatorial explosion including restricting the rules’ applicability domain through further specifications in terms of molecular descriptors (15,16), molecular substructures (17) or chemical similarity (11,18), defining rule probabilities (e.g. through training on BOD (biological oxygen demand) data (19)) and defining rule priorities extracted from collections of known biodegradation pathways as done in EAWAG-PPS (3). All of these approaches have certain advantages and disadvantages (3) but share two important challenges that limit their power: (i) The database for training them is limited, particularly in view of the wide diversity of chemical structures of xenobiotic chemicals, and/or (ii) the data these approaches are trained on are assembled from experiments performed under widely differing conditions and using inocula from diverse sources, without explicit annotation of the meta-information of the conditions.

Hence, the strongly enhanced possibilities in enviPath for users to enter their own data, including all relevant meta-information and to make their data public under a creative commons license will also benefit the further development of these related systems.

Metapath (20) is closely related to enviPath. Metapath is part of the OECD toolbox (see http://www.qsartoolbox.org/) and provides a search engine and database of metabolic maps. Nevertheless, Metapath differs strongly from enviPath. First, Metapath is not freely available on the web. Metapath has to be downloaded and installed, and access is limited via the restrictions of the OECD QSAR toolbox. Additionally, users cannot include their own data sets and are limited to the data provided by the developers. Furthermore, the data in Metapath currently are limited to mammalian biotransformation data, although there are plans to extend those data.

## CONCLUSION AND FUTURE WORK

enviPath is a new system for microbial biotransformation pathways. This system is the successor of EAWAG-BBD, and hence includes the data from EAWAG-BBD, a thoroughly curated and unique data set. Additionally, enviPath provides an easy possibility for the community to share and submit data or to use the expert (rule) and machine learning-based prediction engine to predict new biotransformation pathways. The quality of the data is ensured by a reviewing system that hides data that are not reviewed by a member of the team of reviewers from the user.

Although enviPath already implements a large number of improvements over previous systems, we plan to include more features in the future that expand the scope of the database and improve the prediction accuracy of pathways.

First, we plan to extend and broaden the use of the software itself. One improvement regarding the accessibility of the data is the implementation of a SPARQL endpoint, planned in Version 1.1 (scheduled for mid-2016). Using this endpoint, users can directly query the data they have permissions to read by submitting a SPARQL query. Furthermore, this improvement adds enviPath into the world of linked open data. Other services can then directly link back to enviPath, the same way enviPath links to PubChem, and include data from enviPath directly into other applications or web pages.

Next, we plan to extend the prediction of enviPath to predict not only the biotranformation products, but also the persistence of these products, e.g. the transformation rate of the predicted reactions. Therefore, we will include meta-information on the reactions and environment to improve the predictions. Work on this task is already in progress and is planned to be included in Version 1.2 (scheduled release at the end of 2016).

Another planned feature is the inclusion of data from other databases and the use of these data to improve the models and their predictions. In a first step, we plan to include connections to enzymatic databases and use these data to limit the combinatorial explosion by employing machine learning or by refining the transformation rules. Whereas the inclusion of links to further databases is an ongoing process, and we will update them frequently, the use of these data in the prediction is planned to be included in Version 1.3 (scheduled for 2017).

Finally, we are currently working on making the software more modular and general. Whereas the design already allows an easy maintenance and extension of the software, we plan to extract parts of the software to provide implementations for a wide range of use cases. The easiest modification is to allow for more general metabolic pathway not only limited to biotransformation pathways. Whereas the implementation is easy, gathering the expert knowledge needed to build a basic database is more time consuming. Hence, this is planned to be supported in Version 1.4, scheduled for 2017. We then plan to further split the implementation and provide the system handling user and package management independently from the chemical or pathway part. This can be used for any arbitrary data type, and we are already working on a system for the management and analysis of scientific time series data based on the identical core package (planned again for Version 1.1; scheduled mid-2016).

## References

[1] Kanehisa M., Goto S., Sato Y., Kawashima M., Furumichi M., Tanabe M. (2014). Data, information, knowledge and principle: back to metabolism in KEGG. Nucleic Acids Res..

[2] Kanehisa M., Goto S. (2000). KEGG: kyoto encyclopedia of genes and genomes. Nucleic Acids Res..

[3] Fenner K., Gao J., Kramer S., Ellis L., Wackett L. (2008). Data-driven extraction of relative reasoning rules to limit combinatorial explosion in biodegradation pathway prediction. Bioinformatics.

[4] Wicker J., Fenner K., Ellis L., Wackett L., Kramer S. (2010). Predicting biodegradation products and pathways: a hybrid knowledge- and machine learning-based approach. Bioinformatics.

[5] Wicker J. (2013). Large Classifier Systems in Bio- and Cheminformatics.

[6] Karulin B., Kozhevnikov M. (2011). Ketcher: web-based chemical structure editor. J. Cheminform..

[7] Jeliazkova N., Kochev N. (2011). AMBIT-SMARTS: efficient searching of chemical structures and fragments. Mol. Inform..

[8] Ellis L.B., Gao J., Fenner K., Wackett L.P. (2008). The University of Minnesota pathway prediction system: predicting metabolic logic. Nucleic Acids Res..

[9] Button W.G., Judson P.N., Long A., Vessey J.D. (2003). Using absolute and relative reasoning in the prediction of the potential metabolism of xenobiotics. J. Chem. Inf. Comput. Sci..

[10] Tsoumakas G., Katakis I., Vlahavas I., Maimon O, Rokach L (2010). Mining multi-label data. Data Mining and Knowledge Discovery Handbook.

[11] Moriya Y., Shigemizu D., Hattori M., Tokimatsu T., Kotera M., Goto S., Kanehisa M. (2010). PathPred: an enzyme-catalyzed metabolic pathway prediction server. Nucleic Acids Res..

[12] Dimitrov S., Pavlov T., Dimitrova N., Georgieva D., Nedelcheva D., Kesova A., Vasilev R., Mekenyan O. (2011). Simulation of chemical metabolism for fate and hazard assessment. II CATALOGIC simulation of abiotic and microbial degradation. SAR QSAR Environ. Res..

[13] Finley S.D., Broadbelt L.J., Hatzimanikatis V. (2009). Computational framework for predictive biodegradation. Biotechnol. Bioeng..

[14] Gao J., Ellis L.B.M., Wackett L.P. (2010). The University of Minnesota Biocatalysis/Biodegradation database: improving public access. Nucleic Acids Res..

[15] Embrechts M.J., Ekins S. (2007). Classification of metabolites with kernel-partial least squares (K-PLS). Drug Metab. Dispos..

[16] Mu F., Unkefer P.J., Unkefer C.J., Hlavacek W.S. (2006). Prediction of oxidoreductase-catalyzed reactions based on atomic properties of metabolites. Bioinformatics.

[17] Gómez M.J., Pazos F., Guijarro F.J., de Lorenzo V., Valencia A. (2007). The environmental fate of organic pollutants through the global microbial metabolism. Mol. Syst. Biol..

[18] Oh M., Yamada T., Hattori M., Goto S., Kanehisa M. (2007). Systematic analysis of enzyme-catalyzed reaction patterns and prediction of microbial biodegradation pathways. J. Chem. Info. Model..

[19] Jaworska J., Dimitrov S., Nikolova N., Mekenyan O. (2002). Probabilistic assessment of biodegradability based on metabolic pathways: CATABOL system. SAR QSAR Environ. Res..

[20] Kolanczyk R.C., Schmieder P., Jones W.J., Mekenyan O.G., Chapkanov A., Temelkov S., Kotov S., Velikova M., Kamenska V., Vasilev K. (2012). MetaPath: An electronic knowledge base for collating, exchanging and analyzing case studies of xenobiotic metabolism. Regul. Toxicol. Pharmacol..

